# Diagnostic evaluation of rapid tests for scrub typhus in the Indian population is needed

**DOI:** 10.1186/s40249-016-0137-6

**Published:** 2016-05-12

**Authors:** Siddharudha Shivalli

**Affiliations:** Department of Community Medicine, Yenepoya Medical College, Yenepoya University, Mangalore-575018 Karnataka, India

**Keywords:** India, Rapid test, Scrub typhus

## Abstract

**Background:**

Owing to frequent outbreaks witnessed in different parts of the country in the recent past, scrub typhus is being described as a re-emerging infectious disease in India. Differentiating scrub typhus from other endemic diseases like malaria, leptospirosis, dengue fever, typhoid, etc. is difficult due to overlapping clinical features and a lower positivity for eschars in Asian populations. Hence, the diagnosis heavily relies on laboratory tests.

**Discussion:**

Costs and the need of technical expertise limit the wide use of indirect immunoperoxidase or immunofluorescence assays, ELISA and PCR. The Weil-Felix test is the most commonly used and least expensive serological test, but lacks both sensitivity and specificity. Hence, the diagnosis of scrub typhus is often delayed or overlooked. With due consideration of the cost, rapidity, single test result and simplicity of interpretation, rapid diagnostic tests have come into vogue. However, evaluation of rapid diagnostic tests for scrub typhus in the Indian population is needed to justify or discourage their use.

**Conclusion:**

Research studies are needed to find the most suitable test in terms of the rapidity of the result, simplicity of the procedure, ease of interpretation and cost to be used in the Indian populace.

**Electronic supplementary material:**

The online version of this article (doi:10.1186/s40249-016-0137-6) contains supplementary material, which is available to authorized users.

## Multilingual abstract

Please see Additional file 1 for translations of the abstract into the six official working languages of the United Nations.

## Introduction

Scrub typhus is caused by *Orientia tsutsugamushi* and is transmitted by the bite of infected larvae of the mite *Leptotrombidium deliense*. It is a zoonosis, with humans being accidental, dead end hosts. India is an integral component of the “*tsutsugamushi* triangle” which depicts a part of the globe (northern Japan and eastern Russia in the north, northern Australia in the south, and Pakistan in the west) endemic to scrub typhus. Owing to frequent outbreaks witnessed in different parts of the country in the recent past, it is being described as a re-emerging infectious disease in India. It is one of the most under-diagnosed and under-reported febrile illnesses requiring hospitalization in the region. Eschar (painless, punched out ulcer up to 1 cm in width with a black necrotic centre) is an important finding for the diagnosis of scrub typhus. However, eschar positivity in India and other Asian populations is very low [1]. The absence of an eschar does not rule out scrub typhus in Indian context and hence, the diagnosis relies heavily on laboratory tests.

## Discussion

Scrub typhus is essentially an occupational disease among the rural residents (i.e. farmers, those involved in collecting firewood from the jungle, fishing in the pond etc.) in the Asia-Pacific region [2]. Many tests are available for the diagnosis of scrub typhus and each test has its own advantages and limitations. Indirect immunoperoxidase and immunofluorescence assays are the gold standards for diagnosis [3]. However, the cost and need of technical expertise limit their wide use. Similarly, Enzyme Linked Immunosorbent assay (ELISA) and Ploymerase Chain Reaction (PCR) are not available beyond the secondary level of health care like district hospitals in India [3]. ELISA is easy and relatively economical, but samples need to be pooled, which may delay the diagnosis and affect the overall disease outcome. Many of these tests are not available in remote/rural areas where most cases occur [2]. Weil-Felix test is a commonly used in-expensive serological test that lacks both sensitivity and specificity [4].

Differentiating scrub typhus from other endemic diseases like malaria, leptospirosis, dengue fever, and typhoid is often difficult for a clinician. Lower positivity for an eschar in Asian populations and lack of wide availability of confirmatory tests further augments this problem. In addition, scrub typhus co-infections with other endemic diseases, due to common behavioural risk factors (i.e. outdoor activity or sleeping, lack of personal protective measures and conducive environment for the vector), increases the need for specific diagnosis [5–9].

In such a scenario, role of rapid diagnostic tests is crucial. With due consideration of the cost, rapidity, single test result and simplicity of interpretation, rapid diagnostic tests have come into vogue. The development of rapid diagnostic tests by the use of immunochromatographic test (ICT) technologies has provided a mechanism for point-of-care serological testing [10]. Use and limitations of immunochromatography based rapid tests have been reported from many countries [11, 12]. In China and Thailand, broadly reactive rapid ICTs for scrub typhus have been evaluated by comparing with standard immunofluorescence [13, 14]. These ICTs were more sensitive [13, 14] and specific [14] than the immunofluorescence assay for the early diagnosis of scrub typhus. The absolute sensitivities of currently useful molecular assays in the acute setting are lower (36–56 %) for the diagnosis of scrub typhus [15, 16]. These reports suggest the need of sensitive immunoassays to cover the dynamic spectrum of diagnostic positivity.

In addition, cut offs must be validated locally and local strains should be included in the antigen pool for the effective disease diagnosis owing to antigenic diversity in *O. tsutsugamushi* strains [10, 12]. Newer tests should not to be validated against IFA alone but instead, be compared against a panel of both serological and antigen-detection assays [17]. More emphasis should also be placed on the development of rapid and inexpensive non–serology based means of diagnosis, such as nucleic acid amplification or antigen detection [18].

However, such studies are yet to be reported from India. Recently published DHR-ICMR guidelines for the diagnosis and management of *rickettsial* diseases in India also discourage the use of rapid tests as they need further evaluation [3]. Hence, diagnostic evaluation of scrub typhus rapid tests in Indian populations is imperative to justify or discourage their use.

## Conclusion

Research studies are needed to find the most suitable test in terms of rapidity of the result, simplicity of the procedure, ease of interpretation and cost to be used in the Indian populace.

## Additional file

Additional file 1: Multilingual abstracts in the six official working languages of the United Nations. (PDF 350 kb)

## Abbreviations

ELISA: Enzyme Linked Immunosorbent assay
PCR: Ploymerase Chain Reaction
