# Supplementary material for: Diagnostic evaluation of rapid tests for scrub typhus in the Indian population is needed
Source: Infect Dis Poverty. 2016 May 12;5:40. doi: 10.1186/s40249-016-0137-6 (PMC4865024; doi:10.1186/s40249-016-0137-6)
Supplement: Additional file 1: — Multilingual abstracts in the six official working languages of the United Nations. (PDF 350 kb) [file 40249_2016_137_MOESM1_ESM.pdf]

Translation of the abstract into the five official working languages of the United Nations

الحاجة إلى تقييم تشخيصي للاختبارات السريعة للتيفوس الأكاليّ في المجموعات السكانية الهندية

سيدارودا شيفالي

موجز
الخلفية: نظرا لتعدد حدوث الفاشيات في أجزاء مختلفة من البلاد في الماضي القريب، يوصف التيفوس الأكالي باعتباره مرضا معديا منبعثا في الهند. التفريق بين التيفوس الأكالي وسواه من الأمراض المتوطنة كالملاريا، داء البريميات، حمى الضنك، التيفويد، إلخ صعب بسبب تراكب المظاهر السريرية وقلّة إيجابية الخشارات (التقشرات الجلدية) في المجموعات السكانية الآسيوية. لذا يعتمد التشخيص بشكل كبير على الفحوص المخبرية.
النقاش: التكاليف والحاجة للخبرات الفنية تحدّ الاستخدام واسع النطاق لمقايسات البيروكسيداز المناعية أو التألق المناعي غير المباشرة، مقايسة الممتز المناعي المرتبط بالإنزيم (**ELISA**) و تفاعل البوليميراز المتسلسل (**PCR**). اختبار فيل فيليكس هو الاختبار المصلي الأكثر شيوعا والأقل كلفة، ولكنه يفتقر إلى كل من الحساسية والنوعية. وبالتالي، فإن تشخيص التيفوس الأكالي كثيرا ما يتأخر أو ما يُتجاهل. ومع وضع الاعتبار للتكلفة، والسرعة، نتيجة الاختبارالوحيد وسهولة التفسير، أصبحت الاختبارات التشخيصية السريعة رائجة. على أي حال، هناك حاجة إلى تقييم اختبارات التشخيص السريع للتيفوس الأكالي بين مجموعات السكان الهنود لتبرير عدم تشجيع استخدامها.
الاستنتاج: هناك حاجة للدراسات البحثية للتوصل إلى تحديد الاختبار الأكثر ملاءمة من حيث سرعة ظهور النتيجة، بساطة الإجراء، وسهولة التفسير والكلفة لكي يستعمل بين مجموعات السكان الهنود.

Translated from English version into Arabic by Lina SM, through

TRANSLATORS
WITHOUT BORDERS

印度人群中需开展恙虫病快速检测的诊断评估

Siddharudha Shivalli

摘要
引言：由于过去的几年印度各地多例恙虫病症频发，恙虫病被视作在印度重新出现的传染病。由于疾病重叠的临床表征和在亚洲人群中焦痂更低的阳性率，要从疟疾、钩端螺旋体病、登革热、伤寒等其他地方性疾病中鉴别出恙虫病有一定困难。因此，恙虫病的诊断在很大程度上依赖于实验室试验。
讨论：成本和检测技术的需求限制了间接免疫过氧化物酶法、免疫荧光法、ELISA 法和 PCR 检测的广泛应用。Weil-Felix 检测是最常用且最便宜的血清学检测方法，但其缺乏敏感性和特异性。因此，恙虫病的诊断常被延误或忽视。充分考虑到成本、快速、测试结果的唯一性和说明简单等要素，快速诊断测试已广泛流行。然而在印度，恙虫病的快速诊断测试则需要通过相应评价来证明其可行性或阻止它的使用。
结论：需要研究发现在结果快速性、过程简单性、印度人群中的便于解释和成本多方面最合适的检测方法。

Translated from English version into Chinese by Zhu Yanqi, edited by Yang Pin, through

Nécessité d’une évaluation diagnostique de tests rapides de dépistage de la fièvre fluviale du Japon chez les populations indiennes

Siddharudha Shivalli

Résumé
Contexte : en raison des fréquentes épidémies observées dans différentes régions du pays au cours des dernières années, la fièvre fluviale du Japon est décrite comme une maladie infectieuse réémergente en Inde. La distinction de la fièvre fluviale du Japon d’autres maladies endémiques comme le paludisme, la leptospirose, la dengue, la fièvre typhoïde, etc. est difficile en raison de leurs caractéristiques cliniques communes et de la plus faible positivité aux escarres chez les populations asiatiques. Par conséquent, la pose du diagnostic dépend dans une large mesure de tests de laboratoire.
Discussion : le coût et la nécessité de disposer d’une expertise technique limitent la large utilisation de tests indirects

par immunoperoxydase ou immunofluorescence, ELISA et PCR. Le test de Weil-Felix est le test sérologique le plus couramment utilisé et le moins onéreux, mais il manque à la fois de sensibilité et de spécificité. C'est pourquoi la fièvre fluviale du Japon est souvent diagnostiquée avec retard ou son diagnostic est négligé. Tenant dûment compte de l'importance du coût, de la rapidité, de la nécessité d'obtention d'un résultat à partir d'un seul test et de la simplicité d'interprétation, les tests de diagnostic rapide sont devenus populaires. Une évaluation diagnostique de tests rapides de dépistage de la fièvre fluviale du Japon chez les populations indiennes est cependant nécessaire afin de justifier ou de déconseiller leur utilisation.
Conclusion : des études de recherche sont requises afin de déterminer le test le plus approprié en termes de rapidité d'obtention du résultat, de simplicité de la procédure, de facilité d'interprétation et de coût à utiliser auprès de populations indiennes.

Translated from English version into French by eric ragu, through

Необходимость оценки экспресс-методов диагностики клещевой индийской лихорадки у населения Индии

Сиддхарудха Шивалли

Резюме
Предпосылки: В последнее время в различных регионах Индии участились вспышки клещевой индийской лихорадки, что позволяет вновь говорить о широком распространении в стране этого инфекционного заболевания. Клещевую индийскую лихорадку сложно отличить от других эндемических заболеваний, таких как малярия, лептоспироз, лихорадка денге, брюшной тиф и др., в связи с похожими клиническими признаками и более низкой положительной реакцией при язвенных поражениях у населения Азии. Исходя из этого, диагностика во многом зависит от лабораторных анализов.
Анализ ситуации: Стоимость и потребности в техническом оснащении сужают возможности использования непрямых иммунопероксидазного и иммунофлюоресцентного методов анализа, ИФА и ПЦР. Реакция Вейля-Феликса является самым распространенным и низкозатратным серологическим анализом, но не обладает достаточной точностью и специфичностью, вследствие чего диагноз клещевой индийской лихорадки часто ставится несвоевременно и неправильно. Экспресс-метод лабораторной диагностики становится все более популярным с учетом таких факторов, как стоимость, быстрота, точность результатов простой пробы и простота расшифровки полученных данных. Однако для подтверждения или, наоборот, снижения использования экспресс-метода при диагностике клещевой индийской лихорадки у населения Индии необходимо произвести оценку данного метода.
Заключение: Требуется провести исследование для нахождения наиболее подходящего метода диагностики клещевой индийской лихорадки у населения Индии с учетом таких критериев, как стоимость, быстрота получения результатов, а также простота процедуры и расшифровки полученных данных.

Translated from English version into Russian by Elena Surina, through

Es necesario efectuar una evaluación diagnóstica de pruebas rápidas para el diagnóstico del tifus de los matorrales en la población de la India.

Siddharudha Shivalli

Resumen
Introducción: Debido a los frecuentes brotes que se han observado en diferentes áreas del país en el último tiempo,

el tifus de los matorrales está siendo considerado una enfermedad infecciosa reemergente en la India. Es difícil distinguir el tifus de los matorrales de otras enfermedades endémicas como la malaria, la leptospirosis, el dengue, la fiebre tifoidea, etc., debido a que sus manifestaciones clínicas pueden coincidir y a la existencia de una baja positividad en escaras en la población asiática. Por lo tanto, el diagnóstico depende en gran medida de los análisis de laboratorio.
Discusión: Los costos y la necesidad de conocimientos técnicos limitan la utilización generalizada de ensayos de inmunoperoxidasa o inmunofluorescencia, ELISA o PCR. El examen serológico más utilizado y más económico es la prueba de Weil-Feliz, pero ésta carece de sensibilidad y especificidad. Como consecuencia, el diagnóstico del tifus de los matorrales suele retrasarse o ser ignorado. Debido a los costos, la rapidez, el hecho que se pueden obtener resultados a partir de una sola prueba y la facilidad de interpretación, las pruebas de diagnóstico rápido se han puesto de moda. Sin embargo, es necesario llevar a cabo una evaluación diagnóstica de las pruebas rápidas para el diagnóstico del tifus de los matorrales en la población de la India.
Conclusión: Es necesario efectuar estudios de investigación para encontrar la prueba más idónea en lo que refiere a resultados, simplicidad de procedimiento, facilidad de interpretación y costos, y poder utilizarla en la población india.

Translated from English version into Spanish by Mpgorgone, through

TRANSLATORS
WITHOUT BORDERS
